# Efficacy of a local anesthetic gel infusion kit for pain relief after minimally invasive colorectal surgery: an open-label, randomized clinical trial

**DOI:** 10.1038/s41598-022-22454-z

**Published:** 2022-10-19

**Authors:** Jung Kyong Shin, Heejoon Jeong, Woo Yong Lee, Seong Hyeon Yun, Yong Beom Cho, Jung Wook Huh, Yoon Ah Park, Woo Seog Sim, Hee Cheol Kim

**Affiliations:** 1 Department of Surgery, Samsung Medical Center, Sungkyunkwan University School of Medicine, 81 Irwon-Ro, Gangnam-Gu, Seoul, 06351 Korea; 2 Department of Anesthesiology and Pain Medicine, Samsung Medical Center, Sungkyunkwan University School of Medicine, Seoul, Korea

**Keywords:** Cancer, Colorectal cancer, Pain management

## Abstract

Continuous wound infusion with local anesthesia is an effective method for reducing postoperative pain after laparoscopic colorectal surgery. However, most subcutaneous local anesthesia is delivered through continuous injection, which can be inconvenient for patients. This study compared the effectiveness of postoperative pain relief from the application of a local poloxamer 407-based ropivacaine hydrogel (Gel) to the incision site with continuous infusion-type ropivacaine administration (On-Q) in patients undergoing laparoscopic colorectal surgery. This prospective, randomized, non-inferiority study included 61 patients who underwent laparoscopic colorectal surgery with an incision length of 3–6 cm. All 61 patients were randomly assigned to the Gel group (poloxamer 407-based 0.75% ropivacaine, 22.5 mg) or the On-Q group (0.2% ropivacaine, 4 mg/hour for two days). Postoperative analgesia was induced in all patients with intravenous patient-controlled analgesia (IV-PCA). The outcome measures, which were assessed for 72 h after surgery, included the total amount of fentanyl consumed via IV-PCA (primary endpoint), and the amount of rescue analgesia (pethidine) and postoperative pain intensity assessed using a numeric rating scale (NRS) [secondary endpoints]. The Gel was administered to 31 patients and On-Q was used for 30 patients. There was no significant difference in the total usage of fentanyl between the two groups (Gel group, 1623.98 mcg; On-Q group, 1595.12 mcg; *P* = 0.806). There was also no significant difference in the frequency of analgesic rescue medication use (*P* = 0.213) or NRS scores (postoperative 6 h, *P* = 0.860; 24 h, *P* = 0.333; 48 h, *P* = 0.168; and 72 h, *P* = 0.655) between the two groups. The Gel, which continuously delivers a local anesthetic to operative sites, can thus be considered an effective device for analgesia and pain relief for midline incisions in laparoscopic colorectal surgery.

## Introduction

Postoperative pain is common after most surgical procedures, including laparoscopic colorectal surgery^1^. Currently, multimodal analgesic methods such as preperitoneal infusion, epidural analgesia, and intravenous pain relief are widely used to manage pain related to surgery. Regional anesthetic techniques are often the most effective methods for managing immediate postoperative incisional pain^1–4^. Of these regional anesthesia techniques, continuous wound infusion (CWI) is often used during surgery as an opioid-sparing method for reducing acute postoperative incisional pain and was shown to have many advantages in several studies^1–4^. A systematic review of randomized, controlled trials demonstrated that postoperative wound infusion with local anesthesia could reduce postoperative pain and associated opioid use^5^. These findings were supported by a recent Cochrane database review of the use of CWI as a local anesthetic for postoperative pain after midline laparotomy for colorectal resections in adults^6^.

One method of administering local anesthetic (e.g., ropivacaine) via CWI is the On-Q system (On-Q). In patients undergoing laparoscopic colorectal surgery, On-Q has been shown to control acute postoperative pain and reduce the associated usage of opioid analgesics^3^. Although there are benefits, CWI is associated with certain complications as the process involves the initial insertion of a catheter between the peritoneum and the fascia, followed by removal of the catheter several days after surgery, which can lead to drug leaking out of the catheter and malfunctioning of the On-Q pump^7–10^. Therefore, there is a need for pain control products that are easier to apply as the use of such products could reduce the discomfort and inconvenience of patients after surgery.

This study assessed the efficacy of a local poloxamer 407 (P407)-based ropivacaine hydrogel (hereafter referred to as Gel). After mixing the hydrogel and ropivacaine to maintain a stable local anesthetic. The product, which transforms from a liquid form at room temperature to a gel at body temperature, can be easily applied to wounds (without the need for catheter injection) between the peritoneum and the fascia. The objective of this confirmatory clinical study was to compare the effectiveness of the Gel and On-Q for postoperative pain relief at the incision site in patients undergoing minimally invasive colorectal surgery.

## Results

### Demographic characteristics of the patients

**Figure 1:**
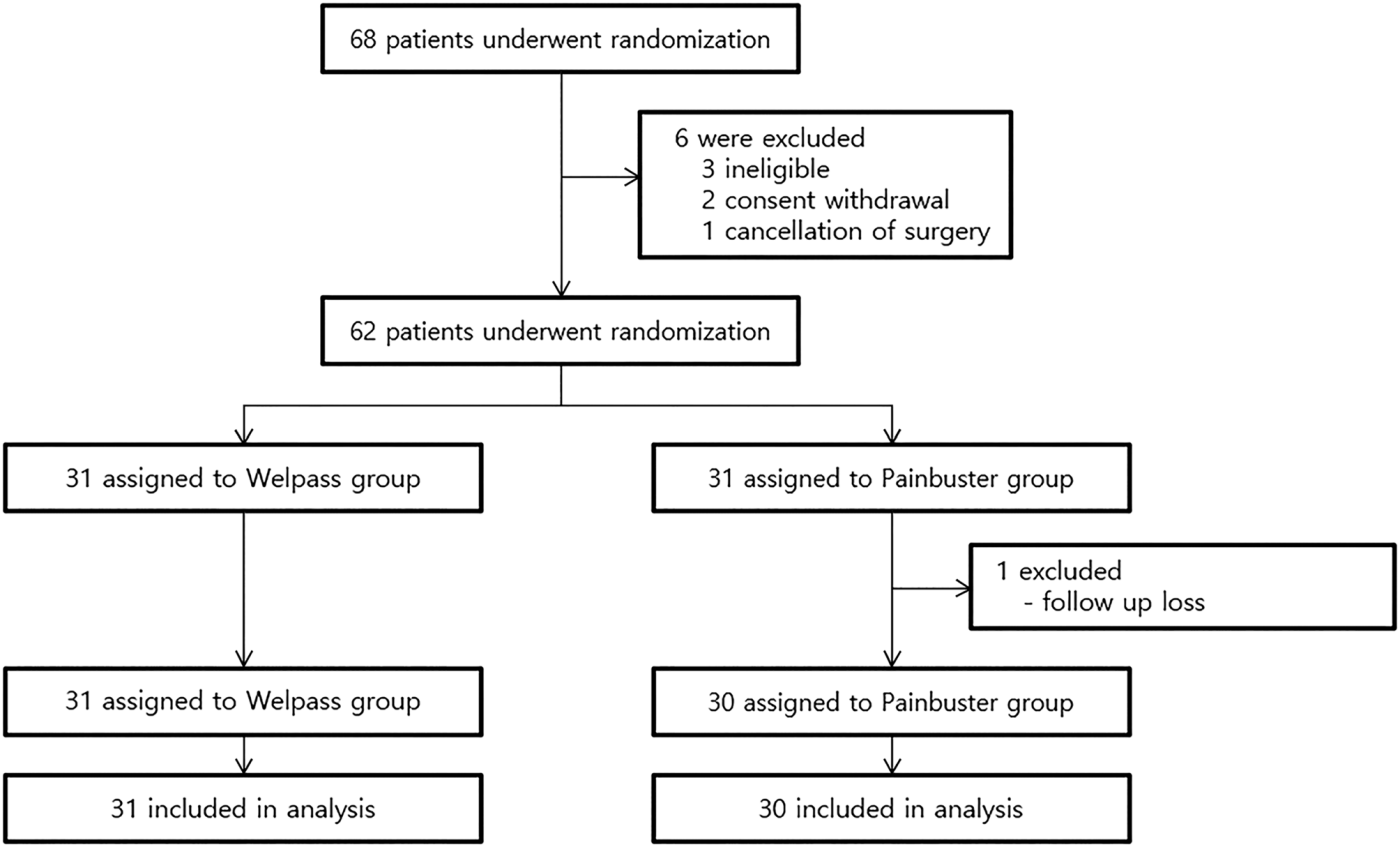
Flow chart of the study.

Figure 1 shows a flow chart of the study. Of 68 patients screened for inclusion in the study, six were excluded from randomization due to ineligibility, the withdrawal of consent, or the cancellation of surgery. Thus, 62 patients were randomized (1:1) to the two study treatment groups. One patient in the On-Q group was excluded from the final analysis because they were lost to follow-up. Consequently, the final analysis was performed with 31 patients in the Gel group and 30 patients in the On-Q group (Fig. 1). Table 1 presents the age, sex, body mass index, and underlying disease distributions between the two groups. With the exception of the median age, which was significantly lower in the Gel vs. the On-Q group (55 vs. 61 years, *P* = 0.036), the two treatment groups were well-balanced.

**Table 1:** Demographic characteristics of patients.

	Gel (*n* = 31)	On-Q (*n* = 30)	*p*
Sex, *n* (%)			0.334
Male	25(80.6)	21(70.0)	
Female	6(19.4)	9(30.0)	
Age, median(years, range)	55(35–72)	61(31–85)	0.036
BMI, median(kg/m^2^, range)	24.5(18.2–29.1)	23.5(16.3–29.3)	0.666
ASA score			0.119
1	8 (26.7)	2 (6.9)	
2	21 (70.0)	25 (86.2)	
3	1 (3.3)	2 (6.9)	
Underlying disease, *n* (%)	12(38.7)	14(46.7)	0.589
DM	2(16.7)	1(7.1)	0.707
HTN	7(58.3)	10(71.4)	0.529
DM/HTN	3(25.0)	2 (14.3)	0.707
Others	0(0.0)	1 (7.1)	0.492
Smoking, *n* (%)			0.382
Current	5(16.1)	5(16.7)	
Former	11 (35.5)	6 (20.0)	
No	15(48.4)	19(63.3)	
Alcohol drink, *n* (%)			0.517
Yes	16(51.6)	13(43.3)	
No	15(48.4)	17(56.7)	
Previous abdominal operation history			0.707
Yes	3(9.7)	4(13.3)	
No	28(90.3)	26(86.7)	

*ASA* American Society of Anesthesiologists, *BMI* body mass index, *DM* diabetes mellitus, *HTN* Hypertension.

### Primary outcome

**Figure 2:**
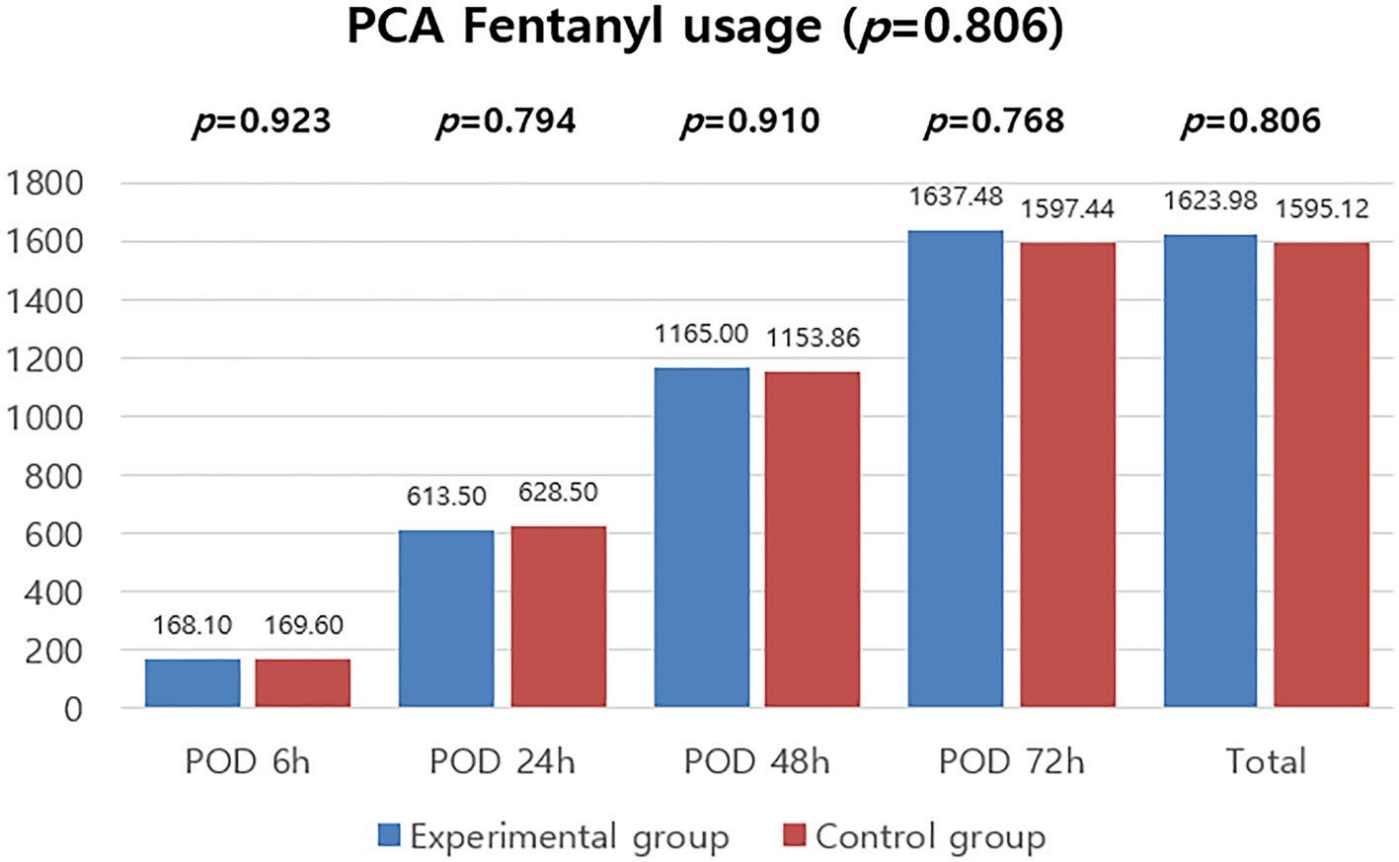
Amount of fentanyl consumed.

To evaluate primary efficacy, we compared total postoperative PCA fentanyl consumption between the two groups of patients. Patients were evaluated for PCA fentanyl consumption after 6 h, 24 h, 48 h, and 72 h. The mean of total postoperative PCA fentanyl consumption values for the Gel and On-Q groups were 168.10 ± 55.25 mcg and 169.60 ± 66.17 mcg at 6 h, 613.50 ± 219.97 mcg and 628.50 ± 225.71 mcg at 24 h, 1165.00 ± 367.60 mcg and 1153.86 ± 368.28 mcg at 48 h, and 1637.48 ± 547.48 mcg and 1597.44 ± 430.81 mcg at 72 h. Based on these data, the total usage was calculated to be 1623.98 ± 500.73 mcg for the Gel group and 1595.12 ± 407.98 mcg for the On-Q group (Table 2). The difference between the results was not statistically significant (*P* = 0.806) (Fig. 2).

**Table 2:** Amount of fentanyl consumption.

	Gel (*n* = 31)	On-Q (*n* = 30)	*p*
Post-operative 6 h (mcg, mean ± SD)	168.10 ± 55.25	169.60 ± 66.17	0.923
Post-operative 24 h (mcg, mean ± SD)	613.50 ± 219.97	628.50 ± 225.71	0.794
Post-operative 48 h (mcg, mean ± SD)	1165.00 ± 367.60	1153.86 ± 368.28	0.910
Post-operative 72 h (mcg, mean ± SD)	1637.48 ± 547.48	1597.44 ± 430.81	0.768
Total usage of fentanyl (mcg, mean ± SD)	1623.98 ± 500.73	1595.12 ± 407.98	0.806

*hrs* hours, *SD* standard deviation.

### Secondary outcomes

**Figure 3:**
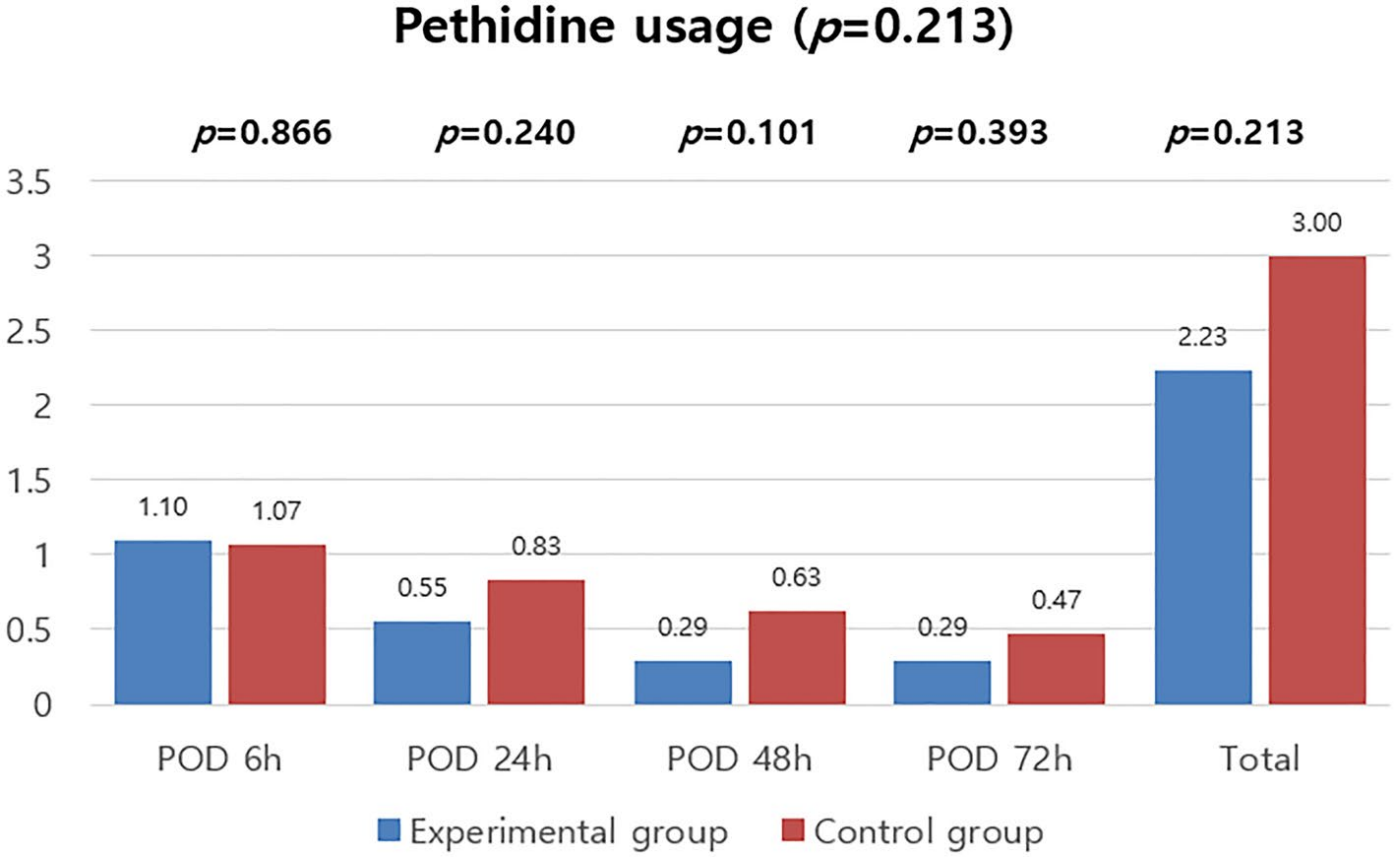
Amount of rescue analgesic consumed.

This study also compared the frequency of using the drug pethidine, which was the secondary outcome. The number of times that patients used the remedy drug was measured at the same times as PCA fentanyl usage mentioned above. The results are shown in Fig. 3. There were no significant differences in the frequency of using pethidine between the two groups.

**Figure 4:**
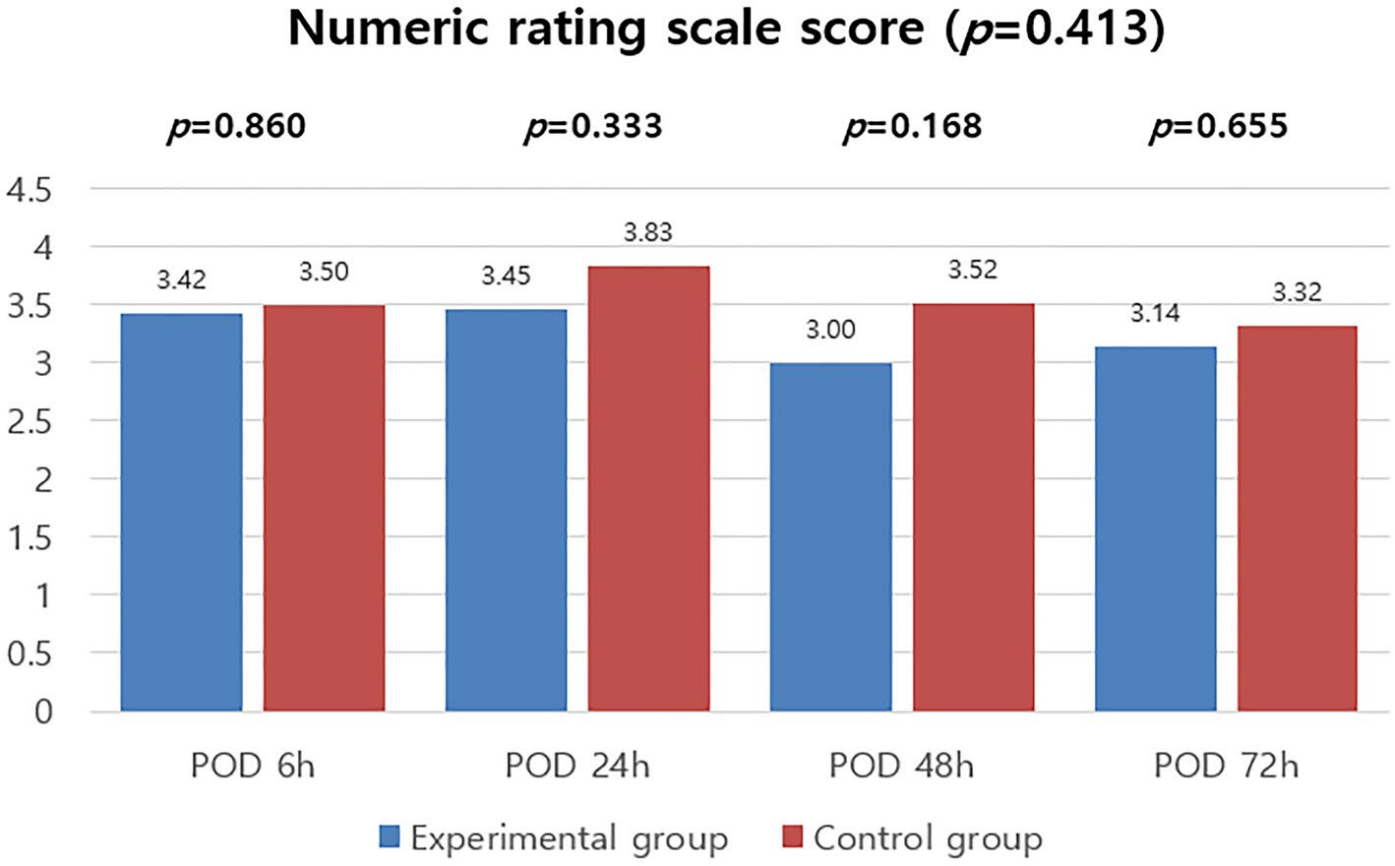
Numeric rating scale scores.

NRS (Numeric Rating Scale) scores were also measured to assess the degree of pain in patients at the same times as the above-mentioned PCA fentanyl usage and pethidine administration frequency were evaluated. A comparison of NRS scores between the two patient groups at each time did not reveal any significant differences. The specific values are shown in Fig. 4.

### Perioperative outcomes

The surgical and postoperative outcomes of the patients in the two groups are reported in Table 3. Most surgeries were performed by a conventional laparoscopic approach. There were no significant differences in the surgical methods used between the two groups (*P* = 0.841). There were no statistically significant differences in other variables such as the location of the primary tumor, operation time, anesthesia time, or estimated blood loss between the two groups. Compared to patients in the Gel group, patients in the On-Q group had a significantly longer mean hospital stay (7.2 ± 1.6 vs. 8.4 ± 2.8 days, *P* = 0.045). One patient in the On-Q group underwent surgery due to chronic constipation with a prolonged hospitalization of 18 days due to postoperative ileus. One patient in the Gel group had a wound seroma, which was treated with a simple dressing in the ward. Any association with the Gel was unclear.

**Table 3:** Perioperative outcomes.

	Gel (*n* = 31)	On-Q (*n* = 30)	*p*
Route of access, *n* (%)			0.841
Conventional LAP	24(77.4)	25(83.3)	
Single port LAP	4(12.9)	3(10.0)	
Robot	3(9.7)	2(6.7)	
Location of primary tumor			0.717
Colon	21(67.7)	19(63.3)	
Rectum	10(32.3)	11(36.7)	
Operation time, median (min, range)	118(58–266)	135(59–229)	0.925
Anesthesia time, median (min, range)	161(89–319)	173(97–272)	0.956
EBL, median (mL, range Range)	50(20–200)	50(10–170)	0.162
Hospital stay days, mean (SD)	7.2(± 1.6)	8.4(± 2.8)	0.045
Postoperative morbidity	2(6.5)	1(3.3)	0.513
Wound seroma	1(3.2)	0(0.0)	0.508
Intraabodminal fluid collection	1(3.2)	0(0.0)	0.508
Postoperative ileus	0(0.0)	1(303)	0.492

*EBL* estimated blood loss, *LAP* laparoscopic surgery, *SD* standard deviation.

## Discussion

To our knowledge, this was the first study to compare the efficacy of Gel (a P407-based ropivacaine hydrogel, the continuous delivery of local anesthetic to operative sites) and On-Q in patients undergoing laparoscopic colorectal surgery. In this study, Gel showed no significant difference in the primary (amount of IV-PCA fentanyl usage up to 72 h postoperatively) and secondary (rescue medication [pethidine] and pain scores) outcomes compared to On-Q. These findings are meaningful from clinical and practical perspectives because the use of On-Q requires the insertion of a catheter, the patient needs to continue to receive the product for some period (typically three days) after surgery, and catheter removal needs to be performed while the patient is still hospitalized. In contrast, the Gel can be applied easily after surgery without the need to mount or remove the product after recovery.

Despite the many advancements in the field of analgesia, postoperative pain remains a considerable concern for patients^11^. Abdominal surgery is particularly known to be associated with postoperative pain due to abdominal incisions^12,13^. Therefore, many analgesic and anesthetic medications have been studied to reduce postoperative pain from abdominal incisions. The continuous infusion of anesthetics is a method that has been applied widely^14–16^, and multiple studies showed that the continuous injection of ropivacaine could reduce postoperative pain^1,17,18^. However, the pain control devices developed in the past have been difficult to apply to patients, which has the disadvantage of prolonging operation times. For example, infusion pumps such as On-Q require training in both assembly and application. Therefore, they generally need to be used by skilled medical personnel.

To overcome these disadvantages and help patients achieve postoperative pain relief, we developed a gel-type infusion kit that could be applied simply and directly to surgical incisions by mixing with local anesthetics. As shown in the results of this study, patients recovering from laparoscopic colorectal surgery using the Gel showed no significant postoperative differences in total PCA fentanyl usage, the amount of rescue medication (pethidine), or the degree of pain compared to patients using the On-Q system. Moreover, the total dose of ropivacaine used in the Gel group was 22.5 mg, which was notably less than the 192 mg dose used in the On-Q group. Thus, we obtained meaningful results in that, compared to On-Q, the Gel could be applied to patients more easily, and it could achieve a similar analgesic effect while using less ropivacaine without inconvenience to the patient due to catheter removal or the discharge of ropivacaine.

The study had several strengths and limitations. First, the nature of the surgical intervention precluded patients and investigators from being blinded to the treatment allocations, which could introduce an element of bias to the study. However, selection bias was minimized through the randomization process. Nevertheless, the median age of the patients in the Gel group was significantly lower than that in the On-Q group, possibly reflecting inadequate randomization. Moreover, the study focused on short-term outcomes associated with acute postoperative pain (e.g., amount of fentanyl via IV-PCA, level of subjective pain, and use of rescue medication); other outcome measures—including time to first bowel movement, ambulation, and quality of sleep and recovery—determined over a longer time would provide a broader picture of the effectiveness of the Gel beyond the immediate 72-h postoperative period. The design of future larger confirmatory studies will incorporate these considerations.

This confirmatory, randomized clinical trial showed that the Gel was at least as effective as On-Q in controlling acute postoperative pain after laparoscopic colorectal surgery while using considerably less ropivacaine. Both products were considered to be safe and well-tolerated. However, as it is convenient to use, the Gel overcomes some of the disadvantages associated with On-Q while reducing patient discomfort after surgery. Therefore, the Gel can be considered to be an effective method for analgesia and pain relief for midline incisions in patients undergoing laparoscopic colorectal surgery.

## Patients and methods

### Patients

Patients who underwent minimally invasive colorectal surgery from March 2018 to December 2018 at Samsung Medical Center, Seoul, Korea, were selected as the subjects of this study. Minimally invasive surgery included conventional laparoscopy, single-incision laparoscopic surgery, and robotic surgery. The inclusion criteria were as follows: patients aged > 19 years, with no clinically significant abnormal findings on preoperative examination, who underwent minimally invasive surgery expected to produce an incision length of 3–6 cm, and who could use a patient-controlled analgesia (PCA) device. Patients with hypersensitivity to (or a history of hypersensitivity to) ropivacaine or other local amide anesthetic agents, those who had local emergency surgery or a septic condition, those who had a mental illness, those who had been on drugs and pain-related medications within one month prior to surgery, and those with severe co-morbidities such as liver cirrhosis, renal failure, or myocardiopathy, were excluded. This study was approved by the Institutional Review Board (IRB) of Samsung Medical Center (IRB No.2018-01-109), and written informed consent was obtained from all subjects participating in the trial. This study was carried out in accordance with all relevant guidelines and regulations. The trial was registered with the Clinical Research Information Service (CRIS) (cris.nih.go.kr; KCT0006079, Hee Cheol Kim, 03/03/2021). This study did not receive research funding from the Ministry of Health and Welfare, so there was no obligation to register with the CRIS before the study. Following the recommendations, we enrolled the clinical study in the CRIS after the start of the study.

### Surgical design

**Figure 5:**
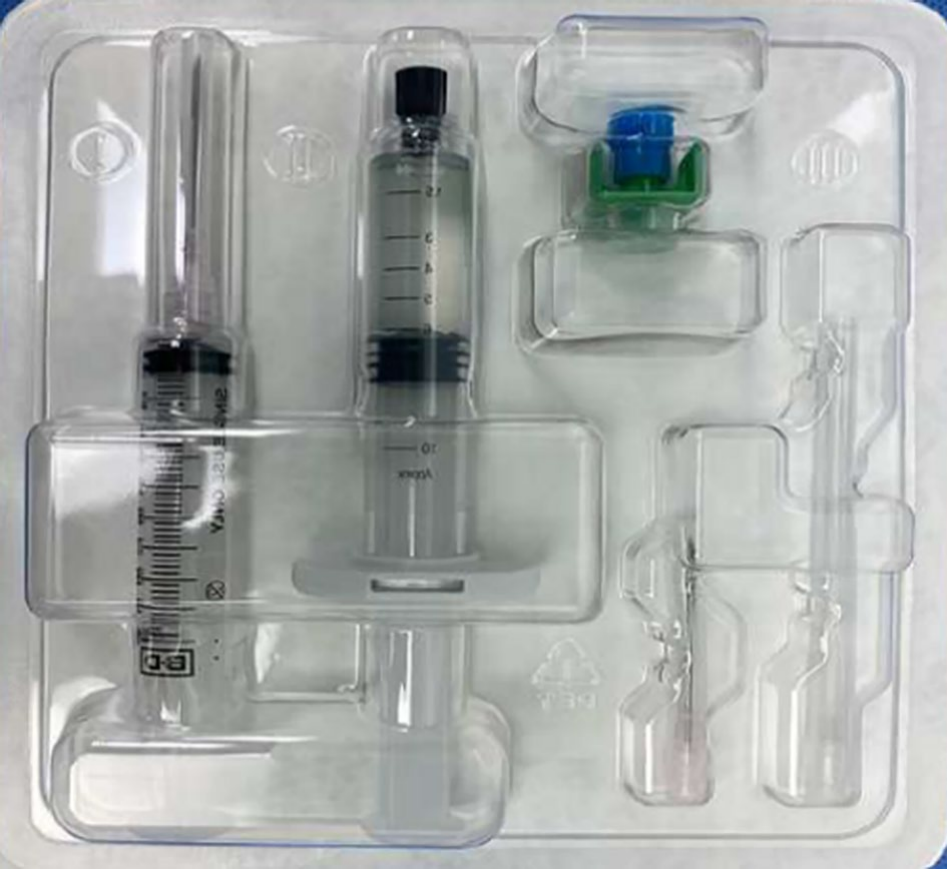
Gel package consisting of gel, connector, and empty syringe for ropivacaine.

**Figure 6:**
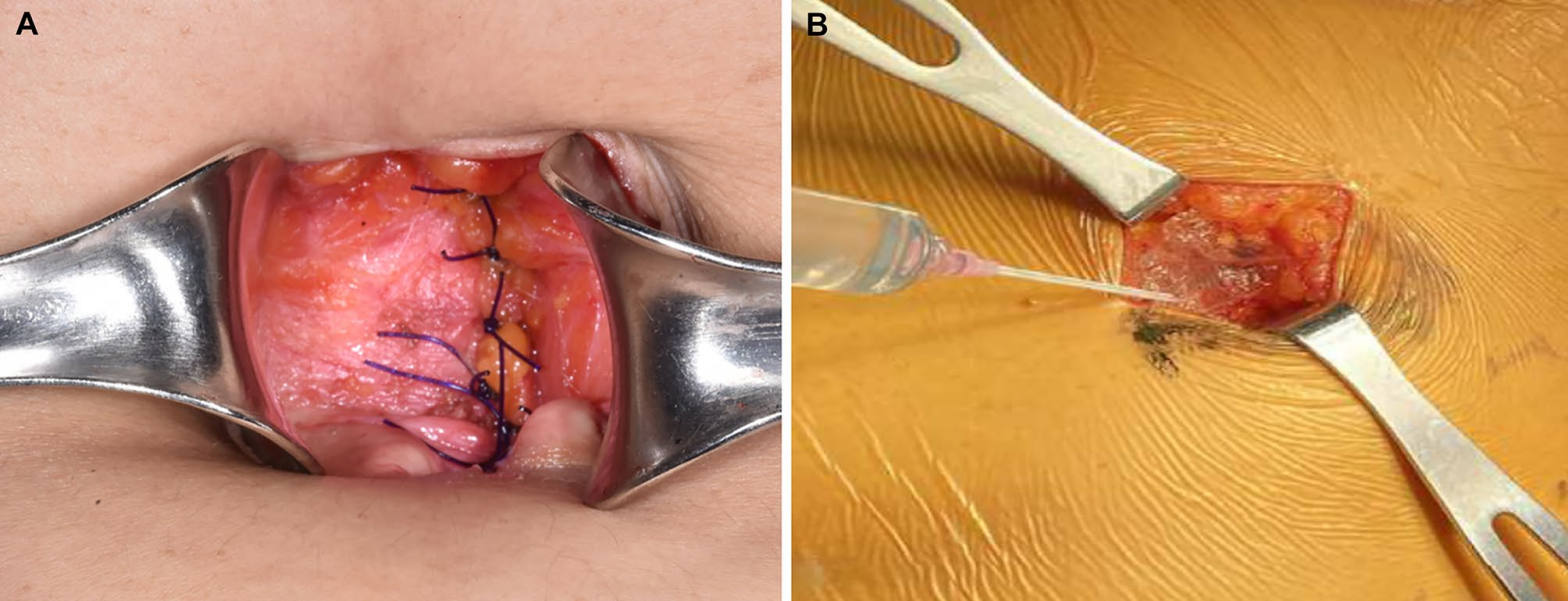
Application of Gel at the wound site.

This was an open-label, parallel, randomized, non-inferiority clinical study. Descriptions of the Gel and the On-Q system, as well as the application techniques, have been reported previously^19^. The application of Gel and On-Q was performed during surgery, in the process of completing intra-abdominal surgery and suturing the wound. The Gel package consisted of one syringe prefilled with gel, one syringe connector, and one empty syringe for ropivacaine (Fig. 5). Initially, the empty syringe was filled with 3 mL of 0.75% ropivacaine, after which this syringe was connected to the syringe prefilled with gel using the connector. Then, 6 mL of a mixture of the gel and ropivacaine was easily obtained by repeatedly pushing each side of the syringe. For the experimental group, poloxamer 407-based ropivacaine hydrogel was applied between the peritoneum and the fascia after mixing 0.75% ropivacaine (3 mL, total dose of 22.5 mg) with 6 mL of the Gel that constituted the product (Fig. 6).

**Figure 7:**
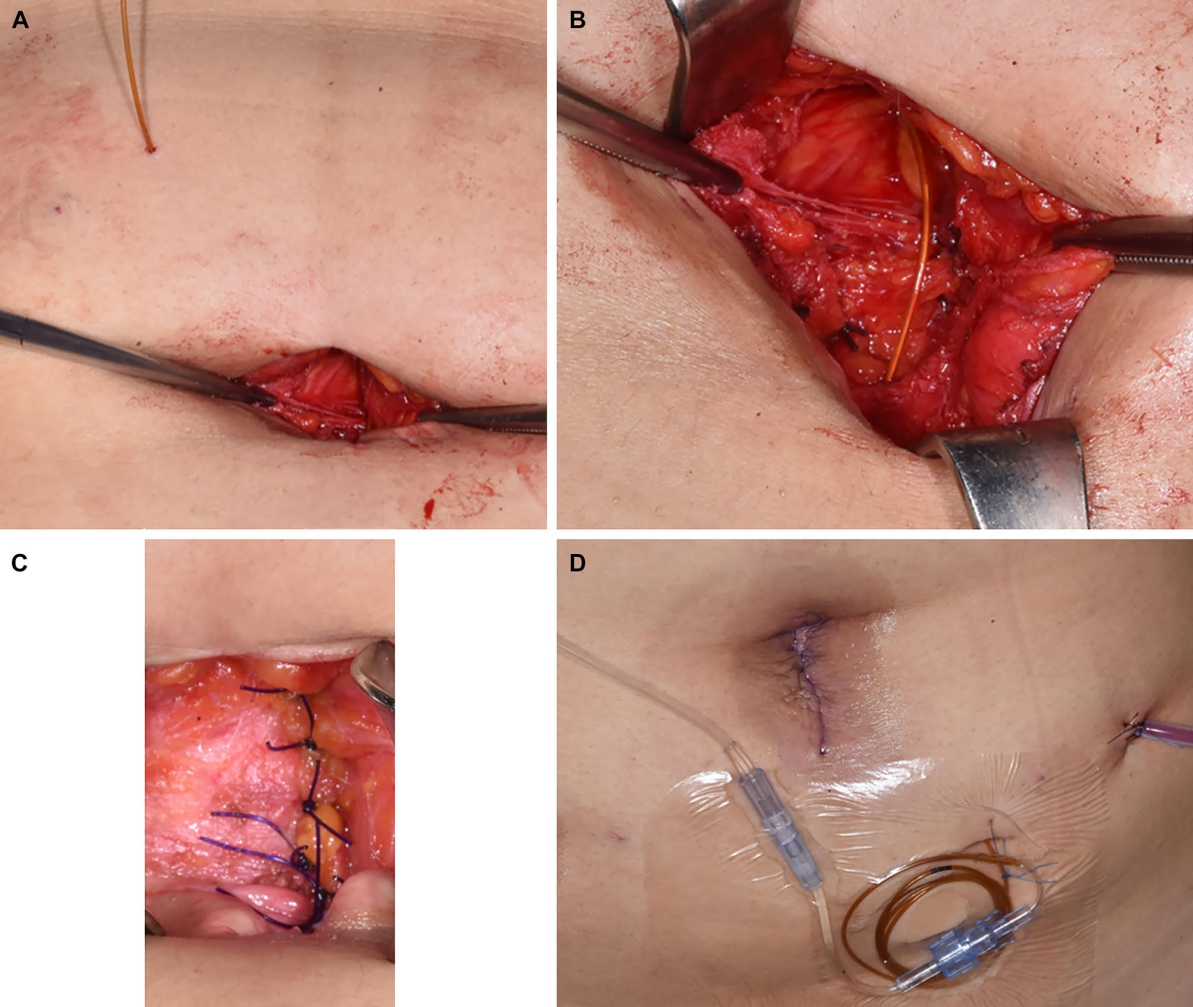
On-Q system procedures.

For the comparator group, the On-Q system consisted of a 25-mm silver-19-coated catheter and a continuous PCA pump. For the control group, the catheter was inserted through a 6.5-cm catheter between the peritoneum and the fascia and connected with an injection pump containing 0.2% ropivacaine (96 mL [flow rate: 2 mL/hour] total dose of 192 mg) (Fig. 7).

Immediately after surgery, both groups received concomitant intravenous PCA (30 mL [1500 mcg] of fentanyl and 70 mL of normal saline), which was delivered via a manual infusion pump (model M1015M, Woo Young Medical Co. Ltd., Chungbuk, Republic of Korea). The total volume of PCA was 100 mL, the flow rate was 1.0 mL/hour, the bolus volume was 1.0 mL, and the lockout time was set at 15 min. In cases where the patient complained of pain (NRS score ≤ 4), rescue medication (pethidine 25 or 50 mg) was allowed up to 72 h postoperatively.

Patients were assigned to either the experimental group (Gel) or the active comparator group (On-Q) according to a randomization table. Randomization (in a 1:1 ratio) was performed to reduce bias that might arise in the allocation of patients and to increase the comparability of the two groups. Block randomization was performed by generating random numbers with four decimal places per person using the Random Function (RAND) in Microsoft Excel (Microsoft Excel 2019 MSO 16.0.10389.20033). Each assignment envelope was sealed and delivered to the investigator. The subject numbers were assigned by the clinical laboratory, and the envelope was opened by the clinical trial coordinator in charge of the study. Patients and investigators were not blinded to the treatment allocations.

### Data collection

The amount of PCA fentanyl used, the amount of rescue analgesic (pethidine) used, and the pain score (NRS; 0–10 scale, with 0 representing no pain and 10 the worst pain imaginable) were assessed at 6, 24, 48, and 72 h postoperatively. Blood pressure, pulse rate, body temperature, complete blood count, prothrombin time, activated partial thromboplastin time, and serum laboratory data (including glucose, blood urea nitrogen, total protein, albumin, bilirubin, aspartate aminotransferase, alanine aminotransferase, and alkaline phosphatase levels) were measured at 48 h, 72 h, and 8 days ± 4 days after surgery to capture the occurrence of any adverse reactions.

### Primary and secondary outcomes

The primary endpoint was the mean total consumption of fentanyl used in IV-PCA up to 72 h after surgery in both treatment groups. The secondary endpoints were the amount of rescue analgesia (pethidine) used as well as pain scores (NRS) 6, 24, 48, and 72 h postoperatively. The occurrence of adverse events was monitored during the admission period. Data on vital signs and laboratory tests were collected and assessed for safety outcomes.

### Safety endpoint

During the admission period, careful monitoring for adverse events was performed. Data on the vital signs and laboratory test results described above were collected and analyzed to evaluate safety.

### Statistical analysis

The study was designed to determine non-inferiority for the primary outcome based on the non-inferiority hypothesis^20^. According to previous studies^3,17,21^, the maximum mean difference in PCA fentanyl usage for 72 h between the Gel and On-Q treatment groups was 409.96 mcg and the mean difference in the total amount of fentanyl used up to 72 h postoperatively between the two groups was 394.80 mcg. The noninferiority margin (197.4 mcg) was conservatively set as one-half of 394.80 mcg. Based on this value, it was calculated that 62 patients were needed, including a dropout rate of 10%, with a type 1 error of 2.5% and a power of 80%.

This study used SPSS for Windows version 27.0 (SPSS, Chicago, IL, USA) for all analyses. The chi-squared test, Fisher’s exact test, independent two-sample t-test, or Mann–Whitney U test (when the normality assumption was not met) were used to analyze differences between the two treatment groups. For multiple comparisons, analysis was performed by repeated-measures analysis of variance (ANOVA), and a post-test was confirmed for point-to-point comparison. The two-group effect test was not statistically significant, so the independent two-sample t-test was additionally performed to compare the two groups at each point in time, and the significance level was corrected using the Bonferroni correction method. Results were considered to be statistically significant when the *p*-value was less than 0.05.

### Research involving human participants and/or animals

This study was approved by the Institutional Review Board (IRB) of Samsung Medical Center (IRB No.2018-01-109), and written informed consent was obtained from all subjects participating in the trial. The trial was registered prior to patient enrollment (cris.nih.go.kr; KCT0006079, Hee Cheol Kim, 03/03/2021).

### Informed consent

This study was approved by the Institutional Review Board (IRB) of Samsung Medical Center (IRB No.2018-01-109), and written informed consent was obtained from all subjects participating in the trial.

## Data Availability

The datasets used and/or analyzed during the current study are available from the corresponding author upon reasonable request.
